# The relationship between spatial resolution levels and quantitative myocardial perfusion

**DOI:** 10.1186/1532-429X-15-S1-O84

**Published:** 2013-01-30

**Authors:** Niloufar Zarinabad, Gilion Hautvast, Marcel Breeuwer, Eike Nagel, Amedeo Chiribiri

**Affiliations:** 1 Division of Imaging Science and Biomedical Engeering, Kings College London, London, UK; 2 Biomedical Engineering,Biomedical Image Analysis, Eindhoven University of Technology, Best, Netherlands; 3 Imaging Systems, MR, Philips Healthcare, Best, Netherlands; 4 Healthcare Incubators, Philips Innovation Group, Eindhoven, Netherlands

## Background

Dynamic contrast enhanced cardiovascular magnetic resonance imaging (DCE-CMR) allows for high-resolution analysis of myocardial perfusion by preserving important spatial information. However high resolution perfusion quantification suffers from the poor signal-to-noise ratio (SNR) of voxel-based data. Previously spatial averaging (segmental or sub-segmental analysis) has been used to improve the SNR. This approach results in reduction of spatial resolution and potentially in a loss of information about the extent, localization and transmurality of ischemia.

This study investigates the relationship between the level of spatial resolution and accuracy of perfusion estimates obtained using different deconvolution methods including Fermi function modelling, ARMA, B-spline basis and exponential basis deconvolution. A new quality of fit analysis method which uses autocorrelation function properties [1] is used here to measure the fraction of remaining un-modelled information (FRI) of the data (i.e. the amount of diagnostic information lost).

## Methods

Perfusion data were obtained from five patients with a dual-bolus technique and 0.075 mmol/kg Gadobutrol (Gadovist, Bayer, Germany) injected at 4 ml/minute followed by a 20 ml saline flush using a Philips Achieva 3T (TX) system, equipped with a 32-channel cardiac phased array receiver coil (Philips, Best, Netherlands). Images were acquired using a saturation recovery gradient echo method (TR/TE 3.0 ms/1.0 ms, flip-angle 15°; effective k-t SENSE acceleration 3.8, spatial resolution 1.2x1.2x10 mm, saturation-recovery delay 120 ms).

First, quantification was performed on high-resolution voxel based data. Then spatial averaging was performed in both transmural and angular direction. In each direction, the total number of grouped voxels (region of interest size) was increased to reduce spatial resolution to 50%, 20% and 10 % of its original value. Analysis of the quality of the fitting procedure was performed on each stage of the process and the obtained results were compared.

## Results

**Figure 1:**
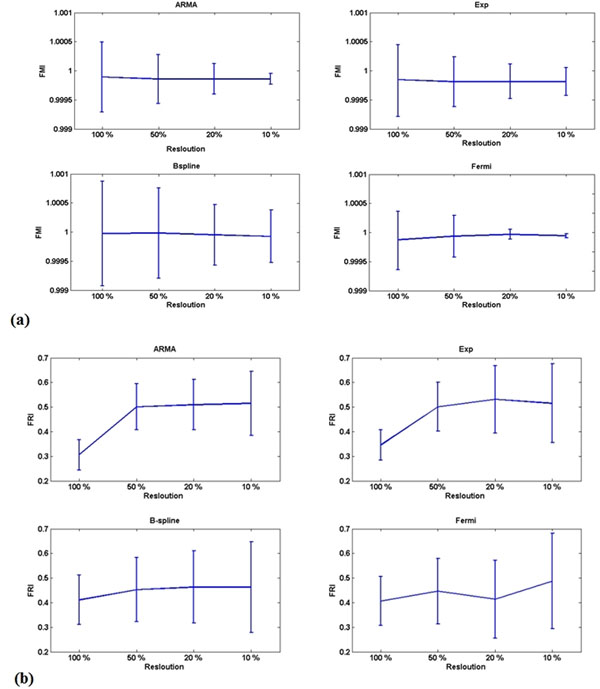
Bars are representing mean and standard deviation of FRI at different level of spatial resolution. Figures (a) and (b) correspond to reduction of the resolution in transmural direction and angular direction, respectively. Mean FRI value has increased as resolution falls down for all methods. This is more obvious for ARMA and exponential methods and transmural direction averaging. STD has increased as resolution falls down for all methods.

The average FRI increased as the resolution was reduced (Fig 1), particularly in the transmural direction. This indicates that increasing spatial averaging is responsible for an increase of the amount of unused information (FRI). This was maintained amongst all deconvolution methods and, in accordance with the expected subendocardial physiological distribution of perfusion defects, was particularly important for spatial averaging in the transmural direction.

## Conclusions

Whilst a reduction in spatial resolution level increases SNR and enables a more stable quantification, a degree of physiological information loss is incurred. Angular direction averaging preserves more information in comparison to transmural averaging preserving potentially useful clinical CMR data.

## Funding

The authors acknowledge financial support from the Department of Health via the National Institute for Health Research (NIHR) comprehensive Biomedical Research Centre award to Guys and St Thomas NHS Foundation Trust in partnership with Kings College London.
